# Structural, Biophysical, and Computational Studies of a Murine Light Chain Dimer

**DOI:** 10.3390/molecules29122885

**Published:** 2024-06-18

**Authors:** Ricardo H. Arriaza, A. Brenda Kapingidza, Coleman Dolamore, Kriti Khatri, Andrea O’Malley, Jill Glesner, Sabina Wuenschmann, Noah P. Hyduke, William Easley, Charline Chhiv, Anna Pomés, Maksymilian Chruszcz

**Affiliations:** 1 Department of Biochemistry and Molecular Biology, Michigan State University, East Lansing, MI 48864, USA; herna921@msu.edu (R.H.A.); khatrikr@msu.edu (K.K.); omalle89@msu.edu (A.O.); 2 Department of Chemistry and Biochemistry, University of South Carolina, Columbia, SC 29208, USA; anyway.kapingidza@duke.edu (A.B.K.); cdolamore2@huskers.unl.edu (C.D.); nhyduke@email.sc.edu (N.P.H.); weasley@email.sc.edu (W.E.); ctchhiv@email.sc.edu (C.C.); 3 InBio, Charlottesville, VA 22903, USA; jglesner@inbio.com (J.G.); sabina@inbio.com (S.W.); apomes@inbio.com (A.P.)

**Keywords:** antibody, dimerization, allergen, crystallography, Der f 1

## Abstract

Antibodies are widely used in medicinal and scientific research due to their ability to bind to a specific antigen. Most often, antibodies are composed of heavy and light chain domains. Under physiological conditions, light chains are produced in excess, as compared to the heavy chain. It is now known that light chains are not silent partners of the heavy chain and can modulate the immune response independently. In this work, the first crystal structure of a light chain dimer originating from mice is described. It represents the light chain dimer of 6A8, a monoclonal antibody specific to the allergen Der f 1. Building on the unexpected occurrence of this kind of dimer, we have demonstrated that this light chain is stable in solution alone. Moreover, enzyme-linked immunosorbent assays (ELISA) have revealed that, when the light chain is not partnered to its corresponding heavy chain, it interacts non-specifically with a wide range of proteins. Computational studies were used to provide insight on the role of the 6A8 heavy chain domain in the specific binding to Der f 1. Overall, this work demonstrates and supports the ongoing notion that light chains can function by themselves and are not silent partners of heavy chains.

## 1. Introduction

In human health, immunoglobulins (Igs) play an important role in the armamentarium against a plethora of pathogens [1]. Whole human immunoglobulin molecules, also known as antibodies, are made up of two identical heavy chains and two identical light chains [1]. Antibody light chains are covalently linked to heavy chains via disulfide bonds, and they exist as two isotypes in humans, kappa (κ) or lambda (λ) [2]. The production of both heavy and light chains is carried out in B cells and, in normal settings, light chains are produced in excess compared to heavy chains [3]. Under physiological conditions, the majority of light chains in the blood serum are bound to heavy chains, and, consequently, the serum level of the secreted unbound light chains (also known as free light chains (FLCs)) is low [4]. Of the two light chain isotypes, lambda FLCs are secreted by plasma cells at a greater rate than kappa light chains, in a ratio of kappa (κ):lambda (λ) of about 1:1.4 [5].

Although FLCs can be found in the serum in higher polymeric forms, monomeric and dimeric light chains are dominant [6]. Several groups have tried to clarify the biological role of FLCs, and their results strongly suggest that these proteins play an independent role in the immune system and could bind antigens [3,7,8]. It is believed that, under physiological conditions, these light chain dimers (LCDs) adopt the canonical quaternary structure of a fragment–antigen binding (Fab) or single-chain fragment variable (scFv) [9]. Nevertheless, a change from this traditional conformation has been linked to light chain pathological disorders, such as light chain amyloidosis, which involves the formation of protein fibers [3,10]. Light chain dimers are also biomarkers of multiple myeloma, a cancer of the plasma cells [11]. These light chain dimers, also known as Bence Jones proteins, are found in the urine of the patients [11]. The regular measurement of FLCs can be used to monitor the progression of diseases, such as multiple myeloma, and to assess the effectiveness of treatments. Changes in FLC levels can indicate how well a patient is responding to the treatment, allowing for adjustments to therapy, as needed. Additionally, FLC levels can also serve as prognostic markers in various blood disorders. High levels of FLCs, or an abnormal kappa/lambda ratio, can be associated with a poor prognosis in multiple myeloma and other related conditions. Interestingly, Bence Jones proteins are not limited to humans, as they have also been found in species that have a similar immunoglobulin structure to humans, which includes many mammals like mice, rats, cows, pigs, and sheep [12,13].

A longstanding goal of our group has been to determine the molecular basis of antibody–antigen recognition in the context of allergies [14,15,16,17]. To achieve this, the recombinant production of antibody fragments, Fab and/or scFv, is needed. This work started with the quest to crystallize the murine IgG1 monoclonal antibody (mAb) 6A8 Fab with its specific antigen, Der f 1, originating from house dust mites and associated with asthma and rhinitis [18]. Der f 1 is a cysteine protease (C1A family) and Group 1 house dust mite allergen. Der f 1 is secreted in the fecal matter of the *Dermatophagoides farinae* mite species. The inhalation of Der f 1 is one of the major risk factors associated with the development of allergic diseases like asthma and atopic dermatitis [19]. This antibody binds to a species-specific epitope on Der f 1, distinct from the epitope bound by the cross-reactive mAb 4C1 that binds to both Der f 1 and Der p 1 allergens [20]. While a crystal structure of 4C1 in complex with Der f 1 has been previously determined by our group (PDB code: 5VPL), a crystal structure of IgG1 Fab 6A8 alone or in complex with Der f 1 has not been elucidated [19,20]. Unexpectedly, the crystal structure of a 6A8 light chain dimer was obtained instead of the intended 6A8 Fab. Building on this unexpected outcome, the biochemical and functional properties of this light chain dimer were explored. Our results have shown that the 6A8 light chain by itself is thermally stable in a wide range of pHs and salt concentrations. Moreover, when the 6A8 light chain is not partnered to its heavy chain, it forms a dimer that lacks Der f 1 specificity and interacts non-specifically with a wide range of allergenic proteins, such as peanut, dog, and cat major allergens, as well as the non-allergenic protein bovine γ-globulin. Consistent with this experimental data, our computational studies suggest that residues from the heavy chain of 6A8 confer Der f 1 specificity by forming a large network of polar interactions with a specific epitope of the allergen. Overall, this has explained the important biochemical and structural differences between the light chain dimer form of 6A8 and the canonical light-chain–heavy-chain dimer. Their distinct characteristics support the ongoing idea that free light chains play a role in modulating the immune system and have implications in the field of antibody applications.

## 2. Results

### 2.1. Crystallization, Structural, and Biochemical Analysis of 6A8 LCD

**Figure 1:**
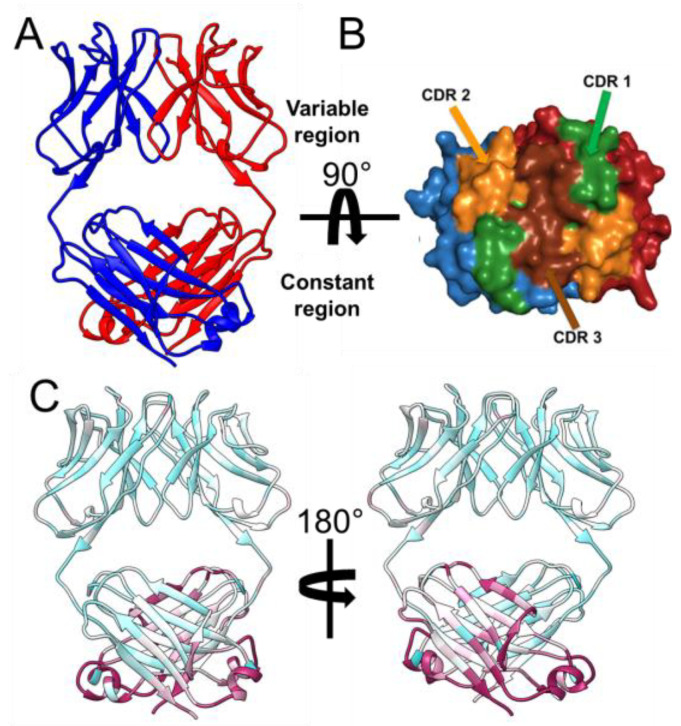
Overall folding and analysis of the 6A8 LCD crystal structure. (**A**) Ribbon representation of the two molecules (red and blue) that comprised the asymmetric unit of this crystal. This dimer is predicted to be stable in the solution, as indicated by PDBePISA analysis. (**B**) Surface presentation of 6A8 LCD, with CDRs labeled. (**C**) Ribbon representation of the average B-factor of each residue. A cyan-maroon scale was used to indicate the lowest to highest calculated B-factors, respectively. Consistent with the interface analysis, the variable region of both chains displays the lowest B-factor values, meaning lowest mobility.

Our initial experiments attempted to express, purify, and crystalize 6A8 Fab (Figure S1A). After exhaustive crystallization efforts, good-quality diffraction data were obtained from what was thought to be a 6A8 Fab crystal. While this structure’s asymmetric unit contained two protein chains, molecular replacement showed that both molecules corresponded to the light chain portion of 6A8. This structure was determined at 2.8 Å resolution (space group: C222, PDB code: 8VY6), and the overall folding resembles the canonical Fab (Figure 1A). In both chains (denoted as A and B), residues 1 to 212 could be modeled. The variable domain expands from residue 1 to 107, and the constant domain is made up of residues 112 to 212 (Figure 1A,B). PDBePISA, a commonly used software that analyzes the quaternary structure of proteins and other macromolecular complexes by evaluating their interfaces, surfaces, and assemblies [21], predicted the dimer observed in the crystal to be stable in solution. Therefore, unexpectedly, the first experimental model of an LCD originated from mice was obtained. Aside from the aforementioned folding, this LCD structure possesses other characteristics expected to be present in a typical Fab. First, the variable and constant domains are composed of the classic antibody β-sandwich folding. Secondly, buried within each, there is an intradomain disulfide bond, formed by C^24^–C^88^ and C^135^–C^195^, in the case of the variable and constant domains, respectively. Interestingly, despite being a homodimer, the conformations of the chains are not identical, with a root-mean-square deviation (RMSD) of 1.2 Å across all single chain Cα atoms. The three complementarity-determining regions (CDRs), which are involved in antigen binding, were predicted for this light chain dimer using the Paratome server (Figure 1B) [22].

PDBePISA predicted this dimer to be stabilized by nine polar interactions, which include seven hydrogen bonds and two salt bridges, and several hydrophobic interactions (Table S1). Among these residues, those that are most buried within the dimeric interface, and thus proposed to play a major role in the stabilization of the quaternary structure, are as follows for chain A: N^35^, L^37^, Q^39,^ I^45^, R^47^, D^56^, L^90^, W^97^, F^99^, and Q^125^; and the following for chain B: N^35^, L^37^, I^45^, R^47^, Y^88^, W^97^, F^99^, S^117^, and F^136^. Interestingly, most of these amino acids belong to the variable domain in each of the two chains. Consistently, the B-factors were lower for the residues located at the variable domains than those at the constant domains (Figure 1C). In addition, PDBePISA calculated a total interface of approximately 1300 Å^2^. The contribution of each domain to this total was calculated by submitting each domain individually to PDBePISA. In this sense, consistent with the idea that the variable domain is driving this dimerization, this region contributed around 750 Å^2^ of this total buried space, while the constant domains are responsible for the remaining 550 Å^2^.

**Figure 2:**
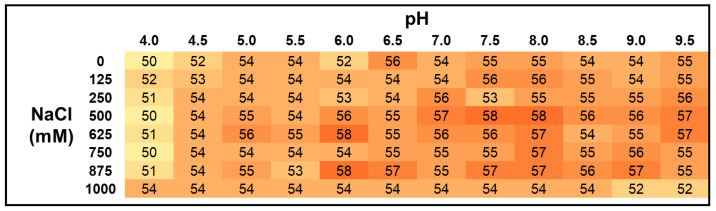
The 6A8 light chain is stable at a wide range of pH conditions and salt concentrations. Thermal stability analysis was performed using DSF in triplicate. This figure displays the average melting temperature of those three independent runs. A two-color gradient going from yellow to orange, lowest to highest, respectively, is used.

Next, we investigated the biochemical behavior of the individual recombinant 6A8 light chain by producing it alone. Upon the purification of the 6A8 light chain (Figure S1B), differential scanning fluorometry (DSF) was used to characterize its thermal stability in a wide range of pH and salt conditions. Except for slightly lower melting temperatures in the most acidic pHs tested (pH 4.0 and 4.5), this experiment revealed that the 6A8 light chain is stable throughout a broad spectrum of pH conditions and salt concentrations (Figure 2).

### 2.2. Comparision of the Quaternary Structure of 6A8 LCD with a 6A8 Fab AlphaFold2 Model and Homologous Light Chain Crystal Structures

**Figure 3:**
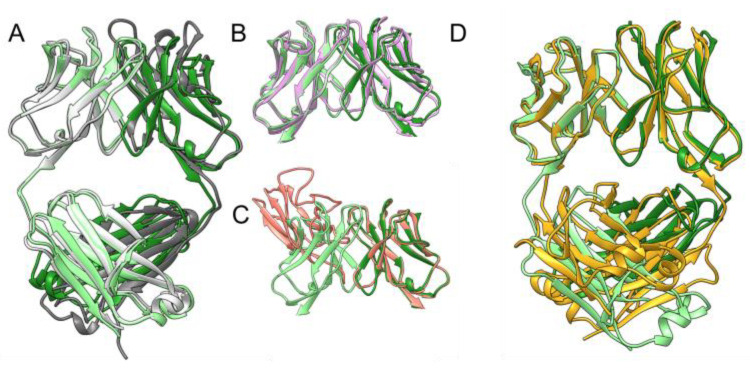
Structural comparison of 6A8 LCD with its corresponding Fab and available V_L_-V_L_ and full light chain dimer structures in the PDB. (**A**) Superimposition of 6A8 LCD (in green) and the AF2 model of its Fab (in gray) highlights the high degree of similarity at the quaternary level. Comparison of the interface between both structures indicated a reduced number of interactions coming from the constant regions in the 6A8 LCD, as opposed to the Fab. The two chains of 6A8 LCD are colored green and light green. The 6A8 AF2 Fab heavy chain is in dark gray, while the light chain is in light gray. (**B**,**C**) Structural comparison of 6A8 LCD with two related V_L_-V_L_ crystal structures. (**B**) Ribbon representation of kappaI O18/O8 light chain germline (in plum) and (**C**) the corresponding amyloid partner, Al-09 (in salmon). These two figures point out the interface difference between the scFv-like fold and the proposed preceding orientation to fiber formation. (**D**) Superimposition between Bence Jones protein DEL (in gold), the only other kappa full light chain dimer available in the PDB, and 6A8 LCD. The variable regions superimpose well among the structures, but the differences in the arrangement of constant domains is quite pronounced.

The molecular basis underlying the conversion of soluble monoclonal light chains into disease-causing fibers remains largely unknown [23]. While the whole light chain can form amyloid fibrils, it is believed that the variable domain plays a major role in driving the aggregation process [24,25,26,27]. As mentioned in the previous section, the 6A8 LCD crystal structure presented the common quaternary structure of a Fab. In fact, the superimposition of this crystal structure with an AlphaFold2 (AF2) [28] model of 6A8 Fab resulted in an overall RMSD of 1.8 Å (across 411 Cα atoms) (Figure 3A). The analysis of the interface between heavy and light chains of the AF2 model revealed that the residues most buried in the oligomeric interface belonging to the heavy chain are as follows: F^107^, L^145^, S^187,^ V^38^, W^48^, F^96^, L^131^, G^146^, L^148^, and S^186^. For the light chain, these residues were as follows: N^35^, L^37^, I^45^, L^90^, F^119^, P^121^, S^132^, V^134^, F^136^, and S^175^ (Table S2). Overall, this interface comparison indicates that, while 6A8 LCD is primarily stabilized by the variable regions, the putative 6A8 Fab is maintained by interactions coming from both the variable and the constant regions. In fact, the total interface area calculated by PDBePISA for the 6A8 AF2 Fab was 1700 Å^2^, and the constant region had a larger contribution than the variable region, being 980 Å^2,^ and 720 Å^2^, respectively, as opposed to the aforementioned interface in the 6A8 LCD crystal structure.

Since the 6A8 LCD crystal structure is the first murine light chain dimer model available in the PDB, we compared the quaternary structure of this crystal structure to other human light chain dimers already deposited. For this analysis, we selected for each protein a single PDB deposit and divided them into two groups, as follows: variable light chain domain dimers (V_L_-V_L_) and full LCD (Tables S3 and S4). Similarly to our 6A8 LCD, the vast majority of the V_L_-V_L_ structures available in the PDB present the characteristic quaternary structure of an scFv, which resulted in low RMSD values across ~200 Cα atoms when superimposed with the variable domain of 6A8 LCD (Table S3). All these V_L_-V_L_ crystal structures, except for a variable domain dimer of an anti-ferritin antibody, are Bence-Jones- and/or amyloidosis-related proteins (Table S3). Moreover, the largest RMSDs arose when 6A8 LCD was superimposed with experimental models with unusual dimer interfaces that were thought to be involved in the aggregation process of these proteins (Table S3). This can be exemplified when comparing the crystal structures of the amyloidosis variable domain Al-09 (PDB code: 2Q1E) and its wild-type (WT) counterpart kappaI O18/O8 germline light chain (PDB code: 2Q20) [10]. The quaternary structure of the experimental model of κI O18/O8 superimposes well with the 6A8 LCD variable domain (Figure 3B), whereas Al-09 presents a 90° rotation from the typical scFv dimeric interface (Figure 3C). Strikingly, a comparison of the quaternary structure of the variable domains of 6A8 LCD and the anti-ferritin V_L_-V_L_ crystal structure also resulted in a high RMSD (22.0 Å across 212 Cα atoms (Figure S2; Table S3)). Nymalm et al. explained how the variable light chain domain can bind human ferritin by itself; however, the domain has different affinity and specificity properties compared to the parent antibody, which they attributed to the distinctive conformation observed in this crystal structure (Figure S2) [29].

When analyzing the full LCDs available in the PDB, we found that all but one, PDB, code: 1B6D (Bence Jones protein DEL), correspond to the lambda isoform (Table S4). Interestingly, the quaternary structure RMSD calculated by the FATCAT server, which allows for flexible superimposition [30] between 6A8 LCD and also a kappa isomer and Bence Jones protein DEL, resulted in one of the highest values, 3.1 Å, but with the lowest number of twists (Table S4). In a similar fashion as 6A8 LCD, the Bence Jones DEL dimer is stabilized by interactions occurring between variable regions [31]. Nevertheless, in the case of this crystal structure, the separation between the constant domains is much more pronounced than that observed in 6A8 LCD (Figure 3D). On the other hand, similarly to the V_L_-V_L_ crystal structures, all but one structure deposited in the PDB of full-length LCDs were classified as Bence Jones proteins and/or linked to light chain amyloidosis. Therefore, our PDB analysis re-emphasizes the uniqueness of the 6A8 LCD crystal structure, as it originates from mice, is the second kappa isoform LCD structure available, and it is not linked, to our knowledge, to any pathological disorders, unlike Bence Jones and amyloid proteins.

### 2.3. Investigating the Specificity and Relative Binding Affinity Properties of 6A8 Light Chain versus the Full 6A8 IgG

Over the years, the idea that the variable heavy chain domain drives the binding to the antigen has been spread [29,32]. Nevertheless, an interesting example that refutes this idea is the variable light chain and full-length light chain dimers produced by mouse myeloma cells, MOPC-315. These dimers are not only capable of binding the antigen derivatives of 2,4-dinitrophenol by themselves, but the variable LCD can also bind other compounds, such as dinitironapthol and menadione, displaying cross-reactivity properties that are not observed in its scFv form [33].

**Figure 4:**
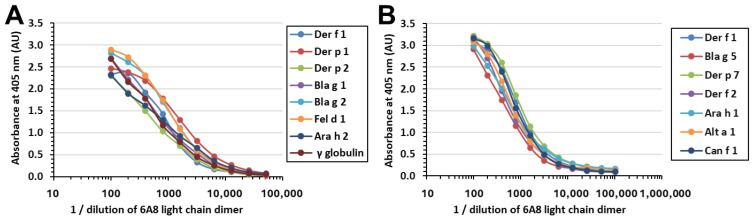
The 6A8 light chain did not show specificity for Der f 1 as the IgG mAb 6A8 does; instead, it displayed non-specific binding towards a wide range of proteins. (**A**) Binding of 6A8 light chain to known allergens originating from house dust mites (Der f 1, Der p 1, and Der p 2), cockroaches (Bla g 1 and Bla g 2), cats (Fel d 1), peanuts (Ara h 2), and the non-allergen bovine γ globulin. (**B**) The binding of 6A8 light chains was proven against additional known allergens from cockroaches (Bla g 5), house dust mites (Der p 7 and Der f 2), peanuts (Ara h 1), fungi (Alt a 1), and dogs (Can f 1).

Given that the 6A8 light chain has been shown to be stable individually, we investigated whether the light chain alone has similar binding specificity as the full-length IgG mAb 6A8 [20]. By employing the enzyme-linked immunosorbent assay (ELISA), it was found that, unlike the complete IgG 6A8 (Figure S3), which is specific to Der f 1 only, the 6A8 light chain interacts with Der p 1 and a wide range of additional allergens (Figure 4A,B). These allergens originate from house dust mites (Der f 2, Der p 1, Der p 2, and Der p 7), cockroaches (Bla g 1, Bla g 2, and Bla g 5), cats (Fel d 1), peanuts (Ara h 1 and Ara h 2), fungi (Alt a 1), and dogs (Can f 1) (Figure 4A,B). Moreover, binding was also detected for the non-allergenic protein bovine γ-globulin (Figure 4A). Given that FLCs are present in circulation, the divergent binding properties between the light chain alone and Fab form are of biological relevance. Furthermore, these data have implications on experiments that employ recombinant versions of Fabs. As shown in this paper, the molecular details obtained by X-ray crystallography were needed to demonstrate that the dimer was actually composed of the homodimer of light chains, rather than a heavy chain and light chain.

### 2.4. Computational Studies to Explore the Difference in Specificity between 6A8 scFv and 6A8 LCD towards Der f 1

Serendipitously, we were not only able to determine the first crystal structure of a light chain dimer originating from mice, but we also demonstrated that the 6A8 light chain, in contrast to the full mAb form, does not specifically bind to Der f 1. Thus, in order to investigate the specific and non-specific binding of the 6A8 mAb and 6A8 light chain dimer, respectively, a computational approach was taken. In this sense, we complemented blind docking studies with epitope and paratope predictions to re-score the blind docking poses obtained. Blind docking allowed the two proteins to interact freely. Given that antibodies tend to have high affinity for their antigen, it was hypothesized that an accurate blind docking program would be able to predict this strong binding, which should be consistent with the epitope–paratope predictions. The details of the method and the reasoning behind it can be found in Section 4.5.

Using the Der f 1 crystal structure (PBD code: 5VPK), the EpiPred server [34] provided three possible epitope regions (hereafter referred to as follows: sandy brown, salmon, and brown, based on the color in Figure S4A. For a complete list of residues forming each epitope, see Table S5). When the 6A8 scFv model and Der f 1 crystal structure were submitted to Antibody i-Patch [35] for paratope prediction, all of the residues that had the highest score and were above the threshold established belonged to the heavy chain domain (Table S6). On the other hand, when the 6A8 LCD crystal structure was submitted to the same server with Der f 1, residues belonging to both light chain molecules were suggested to play a role in antigen binding (Table S6). With this information, the top 10 blind docking poses outputted by ClusPro, employing the antibody–antigen mode [36,37,38], were rescored using AB DockSorter [35], which takes into account the paratope and epitope predictions. For 6A8-scFvin complex with Der f 1, the best pose had a calculated score of 4.6, which was a comparable score to that obtained by our positive control (see Section 4.5). This binding pose presumes that 6A8 scFv recognized the brown-colored epitope (Figure S4B). On the other hand, when the blind docking poses obtained for 6A8 LCD and Der f 1 were rescored, the best pose placed 6A8 LCD interacting with the salmon-colored epitope (Figure S4C). It is important to note that the second-ranked pose of 6A8-scFv complex with Der f 1 was 1.5 points lower than the top-ranked pose. This second pose involved the same epitope. Contrary to this, the second-best ranked pose, 6A8-LCD complex with Der f 1, was only 0.8 points lower than the top one, and it included a different epitope than the top rank, the sandy-brown-colored epitope. Therefore, from the synergistic effect of combining blind docking with epitope–paratope predictions, two main conclusions were found, as follows: First, 6A8 scFv and 6A8 LCD are predicted to bind to different regions of Der f 1 (Figure S5). Second, the difference in score that Ab DockSorter provided among the top 10 blind docking poses suggests that 6A8-scFv–Der-f-1 binding is specific, whereas 6A8 LCD may bind to at least two different regions of Der f 1.

**Figure 5:**
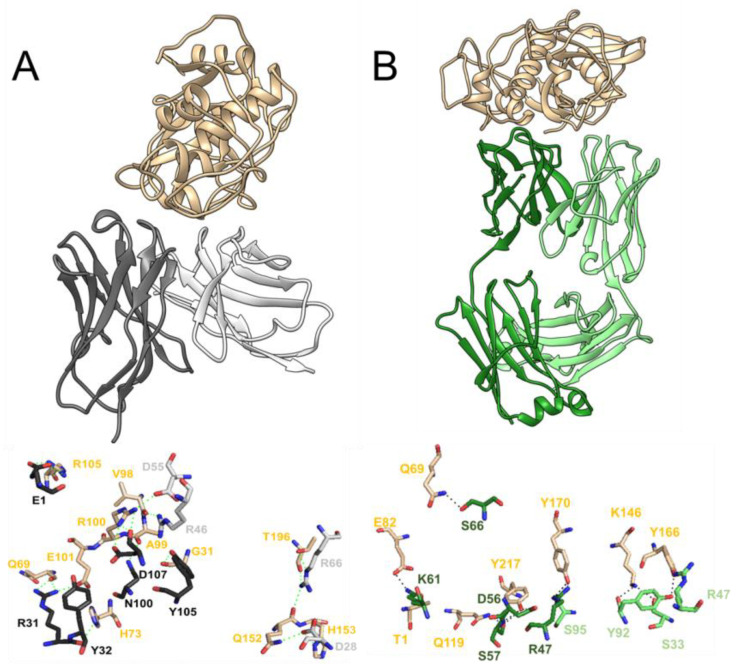
In silico studies of the different forms of binding of 6A8 scFv and 6A8 LCD to Der f 1. (**A**) Proposed mode of binding between Der f 1 (tan color, ribbon representation) and 6A8 scFv (variable light chain in light gray and variable heavy chain in dark gray). On the bottom, the molecular representation of the extensive network of polar interactions (green dashed lines) can be inferred from this pose. (**B**) Ribbon representation of the suggested mode of binding between 6A8 LCD and Der f 1. Molecular view (on the bottom) of the polar interactions (black dashed lines) between light chains and allergens. While in both cases the predicted binding involves several hydrophobic interactions (Tables S7–S10), these results indicate that the large network of polar interactions formed between the variable heavy chain domain and a certain region of Der f 1 underlies the specificity of this binding.

Next, we dissected the interactions taking place in the top rank poses for each case, 6A8-scFv complex with Der f 1 and 6A8-LCD complex with Der-f-1. Starting with 6A8-scFv–Der-f-1, binding appears to be driven by an extensive network of polar interactions. PDBePISA predicted a total of 11 hydrogen bonds and six salt bridges between the variable heavy chain and Der f 1, and six hydrogen bonds and three salt bridges between the allergen and the variable light chain domain (Figure 5A). The interactions from the heavy chain derived from six different residues are as follows: Q^1^, R^31^, Y^32^, N^100^, Y^105^, and D^107^ (Figure 5A); whereas four residues belonging to the light chain are the ones responsible for the polar interactions, as follows: D^28^, R^46^, D^55^, and R^66^ (Figure 5A). Overall, this bonding network involves the following eleven different residues of the allergen: G^31^, Q^69^, H^73^, V^98^, A^99^, R^100^, E^101^, R^105^, Q^152^, H^153^, and T^196^ (Figure 5A). Moreover, several of hydrophobic interactions complement this mode of binding (Tables S7 and S8). Alternatively, when analyzing the top-ranked 6A8-LCD–Der-f-1 pose, a lower number of polar interactions are predicted to be established between the light chains and the allergen. The LCD chain A is presumed to interact through five hydrogen bonds with Der f 1. These involve four residues belonging to the light chain (S^33^, R^47^, Y^92^, and S^95^) and three from Der f 1 (R^146^, Y^166^, and Y^170^) (Figure 5B). The other light chain molecule (chain B) forms six hydrogen bonds and two salt bridges with Der f 1, involving the following: R^47^, D^56^, S^57^, K^61^, and S^66^ from the light chain; and T^1^, Q^69^, E^82^, Q^119^, Y^170^, and Y^217^ from the allergen (Figure 5B). Additionally, several hydrophobic interactions are suggested to contribute to this binding (Tables S9 and S10). Altogether, our results highlight the importance of the variable heavy chain in the specific binding to Der f 1, as it contributes to the formation of a large number of polar interactions, a key type of molecular interaction that drives recognition and specificity [39,40].

It is important to note that the proposed mode of binding between 6A8 scFv and Der f 1 is consistent with the experimentally acquired notion that 6A8 and 4C1 recognized different epitopes of Der f 1 (Figure S6) [19,20]. Furthermore, it allows us to speculate why 6A8 mAb does not bind to Der p 1. Looking at the epitope–paratope interface described in Figure 5A, when superimposing the Der p 1 structure (PDB code: 5VPG.A) with Der f 1, we found two main differences in the residues that comprised this new interface. T^196^ of Der f 1 superimposes with N^195^ of Der p 1. This amino acid substitution is estimated to cause a clash between the R^66^ of the light chain and the N^195^ of Der p 1 (Figure S7), instead of the favorable hydrogen bond previously reported with T^196^ of Der f 1 (Figure 5A). Additionally, P^96^ of Der f 1 superimposes with R^95^ of Der p 1 (Figure S7). While this substitution is not projected to cause any direct non-favorable interactions, such as clashes, the presence of a large, positively charged side chain, as in the case of arginine, may cause a change in the overall chemical environment of the interface, which can interfere with binding.

## 3. Discussion

Immunoglobulins play an indispensable role in the human immune response [41]. Additionally, their remarkable ability to bind to an antigen specifically has led to pervasive use in the scientific and medical fields [42]. Notwithstanding their extensive use, they sometimes perform below par. A recent study involving more than six-hundred commercially available antibodies has demonstrated that hundreds did not perform as expected, either not recognizing their intended target or showing some non-specific binding [43]. Remarkably, this article has concluded that recombinant antibodies performed most effectively [43]. Our work clearly exemplifies how the use of antibodies in various formats can result in unexpected data, even when working with recombinant versions, and highlights the need for rigorous procedures during the purification and identification steps. This will help with the current matter of scientific reproducibility seen in research involving antibodies [44,45,46]. This work also builds on the ongoing notion that free light chains are capable of binding antigens and thus can modulate the immune system, which can lead to non-allergic asthma and rhinitis [3,7,8].

In this work, initial studies involved the recombinant expression and purification of 6A8 Fab. Fabs are composed of heavy chain and light chain domains. While these two chains are very different in terms of sequence identity, their molecular weight tends to be comparable. Therefore, routinely used techniques for the identification of a target protein, such as SDS-PAGE and SEC, are not sensitive enough to distinguish between the aimed heterodimer, heavy-chain–light-chain, and a homodimer, such as light-chain–light-chain. The complexity of the issue is further aggravated if an equilibrium between the heterodimer and the homodimer is considered and possible. The molecular details of the purified product, via crystallographic studies, were necessary to conclude that 6A8 Fab was not being produced.

Our PDB analysis has demonstrated the disparity of structural studies performed to understand the physiological versus pathological roles of light chain dimers. The majority of this type of dimer deposited to the PDB, whether full-length or just variable domain, were obtained in investigations related to the molecular bases of fiber formation and light chain amyloidosis, pathologies that can have detrimental effects on the stomach, large intestine, liver, nerves, and skin [47,48]. In fact, only one of these PDB entries, PDB code: 1F6L, investigates novel antigen-binding properties on an anti-ferritin variable domain homodimer [29]. Contrary to the anti-ferritin V_L_-V_L_, the 6A8 LCD presents the canonical Fab dimer folding and, thus, the structural differences in the dimer interface could not explain the distinct binding properties compared to the Fab form, as it occurs with anti-ferritin V_L_-V_L_. However, these data could be explained by certain experimental conditions (very basic and acidic pHs) used in the ELISA experiments; therefore, under these different conditions, the physiochemical properties of the 6A8 light chain could vary significantly, depending on whether they are interacting with a heavy chain or not, which could underlie the “sticky” features observed with just the light chain in solution [49]. This is consistent with our DSF studies, as the lowest thermal stability that the 6A8 light chain presented was at the lowest pH tested, pH 4.0.

Our computational studies involved two independent programs, ClusPro for blind docking and the specific antibody web server SAbPred [34,36,37,38]. By using blind docking, the system was allowed to interact freely. The hypothesis was that, since antigen–antibody binding tends to be very strong, one among the top poses obtained from blind docking should resemble this tight binding. Since the residues that are responsible for the epitope–paratope interaction in the Der f 1 complex with 6A8-mAb could not be determined experimentally, computational predictions were performed. The accuracy of combining these two programs was initially tested using the 4C1–Der f 1 crystal structure (PDB code: 5VPL) as a positive control. Additionally, the robustness of our system was also tested by carrying out these two experiments independently. For instance, if the epitope and paratope predicted were not seen to interact in any of the docking poses, either the docking program failed or the epitope–paratope predictions were not accurate. Overall, the idea that the heavy chain domain is needed for the specific binding of 6A8 to Der f 1 was supported by our *in silico* studies, as it was predicted to form a large network of polar interactions with the allergen. On the other hand, our *in silico* studies suggest that clashes and changes in the chemical environment of the putative interface may prevent 6A8 mAb from recognizing the similar allergen Der p 1.

In conclusion, our studies present the first model of a murine light chain dimer. These studies are relevant to scientists working with recombinant antibodies, as they suggest the need to perform a thorough identification of purified antibody fragment. In the context of 6A8, this was of particular interest, given that the light chain presented a completely different array of properties related to antigen binding. However, it remains unclear why the light chain dimer crystallized instead of the Fab. It is also not known whether the formation of such a dimer is observed *in vivo*. Assuming that such dimerization occurs in physiological conditions, it may further support the idea that, in the context of the immune system, the light chain is not just a silent partner that is used only for antibody assembly.

## 4. Materials and Methods

### 4.1. Expression and Purification of 6A8 Fab and Light Chain

The DNA sequence corresponding to the light chain variable and constant regions with a C-terminal non-cleavable His-tag was cloned into a pET-22b (+) plasmid, and the heavy chain was cloned into a pET-26b (+) plasmid by Bio Basic (Amherst, NY, USA) [19]. The plasmids were co-transformed into C41 *Escherichia coli* cells. One-liter terrific broth cultures supplemented with kanamycin were grown to OD_600_ of 0.4 at 37 °C and then lowered to room temperature. The cultures were shaken until an OD_600_ of 1.0–1.2 was reached, and then were induced with 500 µM IPTG, cooled to 16 °C, and grown overnight. Cell pellets were resuspended in lysis buffer (50 mM Tris-HCl pH 7.4, 500 mM NaCl, 10 mM imidazole, 2% glycerol) and lysed by sonication. The sonicated mixture was centrifuged at 9000× *g* to obtain a pellet. The supernatant was loaded onto a Bio-Rad Econo-Pac chromatography column equilibrated with 50 mM Tris-HCl pH 7.4, 500 mM NaCl, 30 mM imidazole, and 2% glycerol, containing HisPur Ni-NTA resin. The protein was eluted using an elution buffer of 50 mM Tris-HCl pH 7.4, 50 mM NaCl, 250 mM imidazole, and 2% glycerol. The elution fractions containing protein were determined using SDS-PAGE, pooled, and concentrated using a Millipore Amicon Ultra concentrator (Millipore Sigma, Burlington, NJ, USA) with a 3000 Da molecular mass cutoff. The protein was further purified by SEC using a GE Healthcare ÄKTA-Pure FPLC and Hi-Load Superdex 200 column (Cytiva, Marlborough, MA, USA). The elution buffer was used during the equilibration and during the SEC runs. The same procedure was used for the expression and purification of the light chain alone, but, in this case, only the pET-22 (+) plasmid containing the light chain portion was used for the *E. coli* C-41 transformation.

### 4.2. Crystallization, Data Collection, and Structure Determination of 6A8 LCD

The crystallization of the 6A8 LCD was performed at 277 K. The best diffracting crystals were grown using the sitting drop vapor diffusion method. The crystals were obtained when a 5 mg/mL protein sample was mixed in a 1:1 volume ratio with a well solution composed of 0.2 M ammonium sulfate, 0.1 M Bis-Tris pH 5.5, and 25% *w*/*v* polyethylene glycol 3350. The data collection for the 6A8 LCD was performed remotely using the Southeast Regional Collaborative Access Team (SER-CAT) 22-ID beamline at the Advanced Photon Source (APS), Argonne National Laboratory (Lemont, IL, USA).

The data were processed with HKL-3000 (HKL Research Inc., Charlottesville, VA, USA) [50], and the structure was determined using the molecular replacement method and MOLREP [51] incorporated into HKL-3000. The PDB structure 3RVU was used as a search model. The model was rebuilt with Buccaneer [52] and ARP/wARP [53]. Model refinement was performed using REFMAC and HKL-3000. Some programs from the CCP4 package were used for data handling [54]. The model was manually adjusted using COOT [55]. COOT and MOLPROBITY [56] were used for model validation. TLSMD [57] was used for the determination of the TLS groups used during refinement. The model and structure factors were deposited to the PDB with accession code 8VY6. The data collection, refinement, and validation statistics are summarized in Table S11.

### 4.3. Thermal Stability via DSF

The DSF experiments were performed as previously reported [58]. In brief, 1 mL of 1 mg/mL protein was incubated with 1 µL of SYPRO Orange dye (Thermo Fischer, Waltham, MA, USA) on ice for ten minutes. Then, 10 µL of the protein was mixed with 10 µL of the buffer screen on a Bio-Rad Hardshell 96-well RT-PCR plate and sealed with Bio-Rad Microseal PCR-plate-sealing film (Hercules, CA, USA). Sodium acetate buffer was used for pH 4.0–5.0, Bis-Tris for pH 5.5–6.5, Tris for pH 7.0–8.0, and CHES for pH 8.5–9.5. All experiments were performed in triplicate, with 0.5 pH increments and sodium chloride concentration ranging from 0 to 1.0 M. The temperature at which the protein unfolds was measured as an increase in fluorescence (emission at 590 nm; excitation at 488 nm) with a Bio-Rad CFX96-RT-PCR instrument (Hercules, CA, USA) within the range of 30–90 °C, with 1 °C/min increase in 2 °C increments.

### 4.4. ELISA Experiments: Specificity and Binding Affinities of mAb 6A8 LCD and Full IgG Ab

For the two-site immunoassays, microplates were coated overnight with the murine IgG mAb 4C1 (which cross-reacts with Der f 1 and Der p 1) or 1D8 (Der p 2-specific) at 1:1000 dilutions in 50 mM carbonate/bicarbonate buffer, pH 9.6. The plate was washed and blocked for 1 h with phosphate buffered saline, pH 7.4, containing 0.05% Tween 20 and 1% bovine serum albumin. rDer f 1 and rDer p 1 were added at a concentration of 250 ng/mL, and rDer p 2 at 100 ng/mL. After the 1-h incubation period, 6A8 LCD (1.7 mg/mL) was added at 1:100 and diluted 1:2 across the plate, followed by the addition of biotinylated anti-His-Tag Ab (1:500) (R&D Systems, Minneapolis, MN, USA) and streptavidin–peroxidase (1:1000) (Sigma-Aldrich, St. Louis, MO, USA). The plates were developed using 2,2′-azino-bis (3-ethylbenzothiazoline-6-sulphonic acid) (ABTS) in 70 mM citrate phosphate buffer, pH 4.2, and 1:1000 dilutions of H_2_O_2_. The absorbance was read at 405 nm with a Bio-Tek EL800 Microplate Reader (Figure S8) (Bio-Tek Instruments, Inc., currently Agilent Technologies, Inc., Santa Clara, CA, USA).

To further assess 6A8 LCD specificity, immunoassays were performed to measure the direct binding of 6A8 LCD to thirteen different proteins, including recombinant allergens, expressed in *E. coli* or *Pichia pastoris*, from house dust mites (rDer f 2, rDer p 1, rDer p 2, and rDer p 7), cockroaches (rBla g 1, rBla g 2, and rBla g 5), cats (rFel d 1), peanuts (rAra h 2), fungi (rAlt a 1), and dogs (rCan f 1). Additional proteins included natural Ara h 1, purified from blanched peanuts, and a non-allergenic protein (bovine γ-globulin) (Thermo Scientific, Rockford, IL, USA). A microplate was coated overnight with the allergen or γ-globulin at 10 µg/mL, and blocked the following day, as described above. The 6A8 LCD was added at 1:100 and diluted at 1:2 across the plate. A 1 h incubation of biotinylated anti-His-Tag Ab (1:500) (R&D Systems, Minneapolis, MN, USA) followed, along with a 30 min incubation period with streptavidin–peroxidase (1:1000) (Sigma-Aldrich, St. Louis, MO, USA). The plate was developed as described above.

The relative binding affinity and specificity of mAb 6A8 LCD was compared to the binding and specificity of the full IgG mAb 6A8. Similar ELISA steps as those performed for the light chain dimer were followed for the full antibody; however, the coating of the microplate was carried out with mAb 6A8 1:1000, then nDer f 1 and nDer p 1 at 250 ng/mL (top of curve), and diluted at 1:2 across the plate, followed by B-4C1 at 1:1000. Streptavidin–peroxidase and ABTS/H_2_O_2_ were then added. The plate was developed as elucidated above.

### 4.5. Computational Studies on Protein–Protein Interactions

We combined two independent computational programs to provide insight on the difference in the binding of Der f 1 to 6A8 scFv or its light chain dimer. Although this approach has already been shown to increase the accuracy of obtaining a near-native pose from blind docking [59], we tested the robustness of the approach in the context of allergen–antibody binding, which is a known complex system. For instance, as previously mentioned, 6A8 is specific to Der f 1 and does not recognize Der p 1, despite these two allergens sharing 81% sequence identity and a highly similar overall folding. To achieve this, we chose 4C1, which can be considered our positive control, and 10B9, our negative control. The 4C1 binds to Der f 1, and the molecular basis of this interaction has been determined by crystallographic studies [15]. On the other hand, 10B9 is specific to Der p 1, but it does not recognize Der f 1 [16]. Our working hypothesis was that our approach would be able to recreate 4C1–Der f 1 (PDB code: 5VPL), whereas the score for the blind docking poses of 10B9–Der f 1 would be lower than the aforementioned amount. A scheme explaining this approach is shown in Figure S9.

First, the experimental model of Der f 1 and 4C1 Fab (PDB code: 5VPL) were submitted to ClusPro, using the antibody–antigen mode [36,37,38]. In parallel, EpiPred and Antibody i-Patch, both tools of SAbPred, were used to predict the epitope and paratope residues [34]. While EpiPred outputs three possible epitope regions, Antibody i-Patch gives a score based on the confidence level of a particular residue belonging to the paratope region (the more confident the program, the higher the value). Since all residues from 4C1 that were experimentally determined to be in the paratope region had a predicted score of ≥10, this was established as the cut-off point to consider a residue part of the paratope, or not, for all experiments. Upon obtaining the blind docking results and the epitope and paratope predictions, Ab DockSorter [59] was used to re-score the top 10 docking poses based on the 3 different epitopes and the single paratope predictions. This program gives a score for each pose entirely based on the predicted interactions between a provided list of residues that form the epitope and paratope. The higher the score, the more these two regions are seen to interact in a particular docking pose. The best scoring pose for 4C1 and Der f 1, with a score of 4.7, highly resembled the crystal complex, with an RMSD of 2.1 Å across all 650 Cα atoms. Subsequently, the same model of Der f 1 was submitted to ClusPro, along with 10B9 Fab (PDB code: 4POZ). The highest scoring pose per Ab DockSorter was scored at 3.2, which is almost 40% lower than our positive control.

Overall, these results suggest that, when investigating 6A8–Der f 1 binding, a score close to our positive control suggests a tight, specific binding, whereas lower-scored poses may imply the binding is weaker and, thus, may either be non-specific or may not occur in the real biological system. Following the same procedure as that just described, the binding between 6A8 scFv, modeled with the antibody structure modeling tool from SAbPred [34], and Der f 1 was compared to the 6A8 LCD crystal structure with the same allergen.

### 4.6. Additional Computational Tools

COOT, PyMOL [60], and UCSF-Chimera [61] were used to visualize and analyze the structures, as well as to generate figures. AlphaFold2 was used to model the full-length 6A8 Fab [28]. PDBePISA [21] was used to analyze the interfaces. FATCAT was used for flexible superimposition between the 6A8 LCD and the available Fab in the PDB [30].

## Data Availability

Datasets are available upon request from the authors.

## Supplementary Materials

The following supporting information can be downloaded at: https://www.mdpi.com/article/10.3390/molecules29122885/s1. Figure S1: Size-exclusion chromatography (SEC) profiles and SDS-PAGE for the 6A8 Fab construct; Figure S2: Comparison of the quaternary structure of 6A8 LCD and anti-ferritin VL-VL crystal structure; Figure S3: ELISA binding assays of mAb 6A8 IgG to Der f 1 and Der p 1 allergens (mAb 6A8 is specific to Der f 1); Figure S4: Computational studies that combine epitope–paratope prediction and blind docking; Figure S5: Superimposition of best 6A8-scFv–Der f 1 and 6A8-LCD–Der f 1 docking poses, after rescoring; Figure S6: Superimposition of 6A8-scFv–Der-f-1 best docking pose and 4C1-Fab–Der f 1 crystal structure. Figure S7: Superimposition of Der p 1 structure (plum color) with the Der f 1 from the top-rank docking pose provides insight on the inability of 6A8 to bind to Der p 1; Figure S8: ELISA Binding Assays of 6A8 light chain dimer to Der f 1 and Der p 1; Figure S9: Scheme of the computational approach taken to obtain a robust model of the different protein-protein interactions; Table S1: PDBePISA output for the 6A8 LCD crystal structure used for the analysis of interactions present among the two molecules in the asymmetric unit; Table S2: PDBePISA output for 6A8 AF2 Fab used for the analysis of the interactions between heavy and light chains from this theoretical model; Table S3: Structural comparison of VL-VL crystal structures available in the PDB with the variable region of the 6A8 LCD crystal structure; Table S4: Structural comparison of light chain dimers available in the PDB and 6A8 LCD crystal structure; Table S5: List of residues forming the three different epitopes predicted by EpiPred, named after the colors used in Figure 4A; Table S6: List of residues predicted to be part of the paratope of the 6A8 scFv and 6A8 LCD involved in the binding to Der f 1; Table S7: PDBePISA output analyzing the interactions between the variable heavy chain the domain of 6A8 scFv and Der f 1 from the top-ranked pose; Table S8: PDBePISA output analyzing the interactions between the variable light chain domain of 6A8 scFv and Der f 1 from the top-ranked pose; Table S9: PDBePISA output analyzing the interactions between chain A of 6A8 LCD and Der f 1 from the top-ranked pose; Table S10: PDBePISA output analyzing the interactions between chain B of 6A8 LCD and Der f 1 from the top-ranked pose; Table S11: Summary of data collection, refinement, and model validation statistics.
