# Supplementary material for: Structural, Biophysical, and Computational Studies of a Murine Light Chain Dimer
Source: Molecules. 2024 Jun 18;29(12):2885. doi: 10.3390/molecules29122885 (PMC11206851; doi:10.3390/molecules29122885)
Supplement: Supplementary file 1 [file molecules-29-02885-s001.zip › Supporting figures.pdf]

Supporting figures:

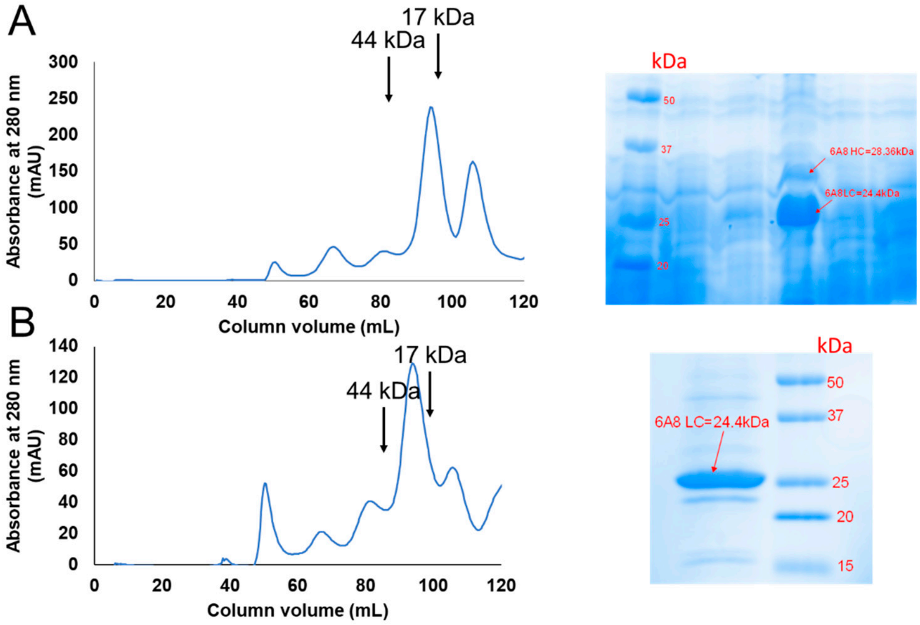

Figure S1. Size-exclusion chromatography (SEC) profiles and SDS-PAGE for 6A8 Fab construct (A) and 6A8 light chain only (B). The apparent molecular weight of each peak observed in the chromatogram was approximated based on standards from Bio-Rad (Hercules, CA), with estimated elution volumes shown for two more relevant for this study.

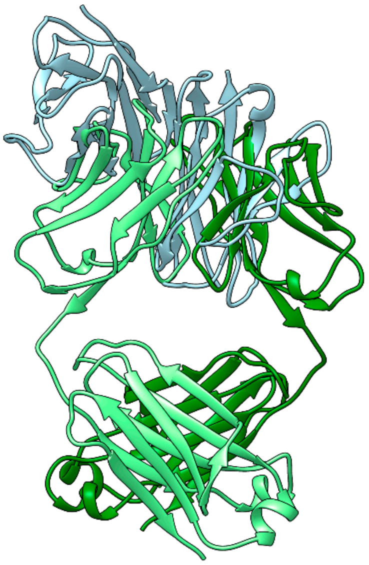

Figure S2. Comparison of the quaternary structure of 6A8 LCD and anti-ferritin VL-VL crystal structure. The superimposition of quaternary structures revealed that the latter one does not present the canonical scFv interface.

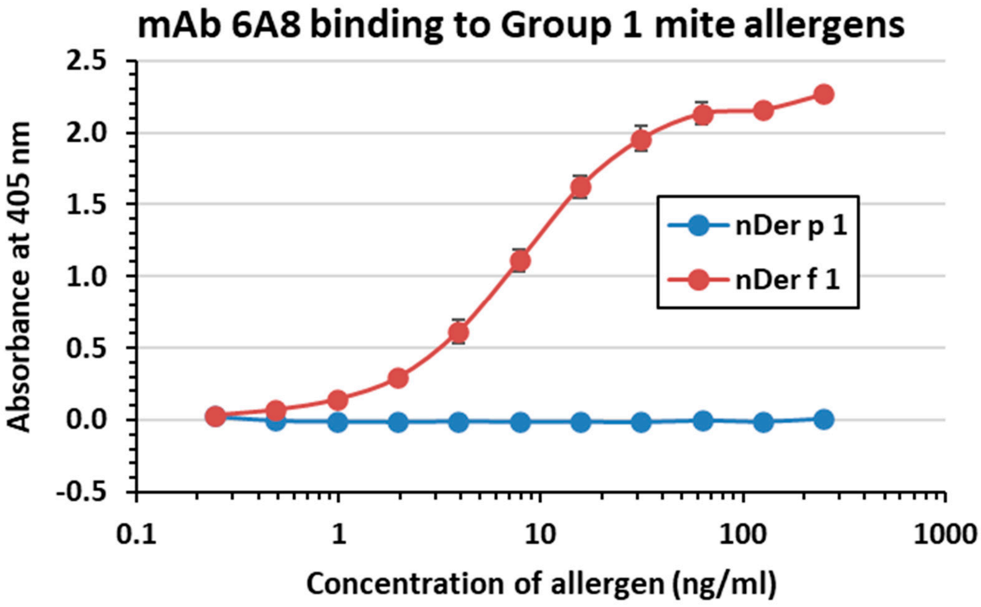

Figure S3. ELISA Binding Assays of mAb 6A8 IgG to Der f 1 and Der p 1 allergens. mAb 6A8 is specific to Der f 1. Data are duplicates ± SD.

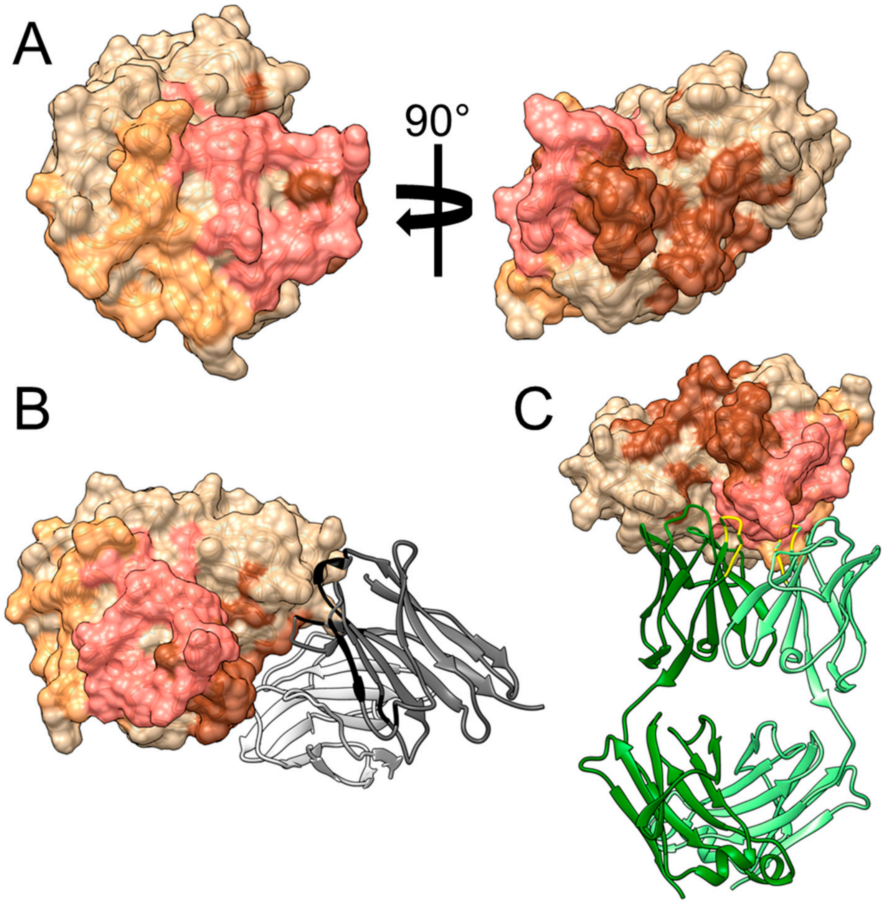

Figure S4. Computational studies that combine epitope – paratope prediction and blind docking. (A) Surface representation of Der f 1 with the three epitopes predicted by EpiPred in three different colors: sandy brown, salmon and brown. Refer to Table S5 for the list of residues for each case (B) Ribbon and surface representation of the best 6A8 scFv and Der f 1 pose after rescoring. In this case, 6A8 scFv is binding to the brown color epitope. The heavy chain of the scFv is shown in dark gray while the light chain is in light gray. Residues predicted to form the paratope of this scFv are colored black. See Table S6 for the list of residues. (C) Representation of 6A8 LCD and Der f 1 best pose. 6A8 LCD is predicted to bind to the salmon-colored epitope, which is different than the region presumably recognized by 6A8 scFv. The residues of 6A8 LCD that formed the paratope are colored yellow. For the full list see Table S6.

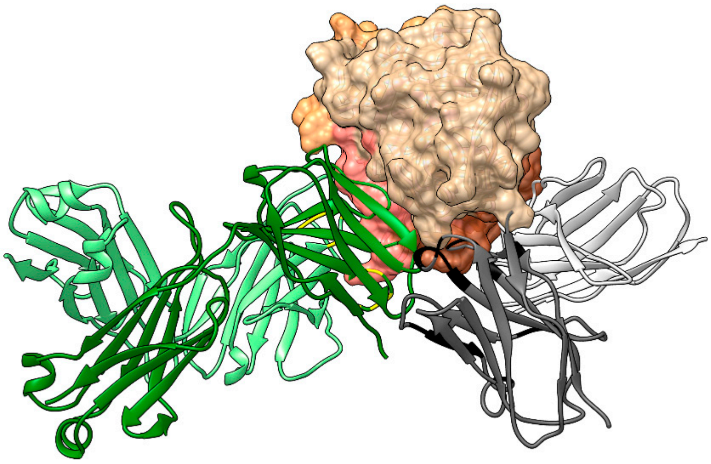

Figure S5. Superimposition of best 6A8 scFv- Der f 1 and 6A8 LCD- Der f 1 docking poses, after rescoring, show how these two are predicted to recognize different epitopes of this allergen. Coloring follows the same scheme as Figure 5.

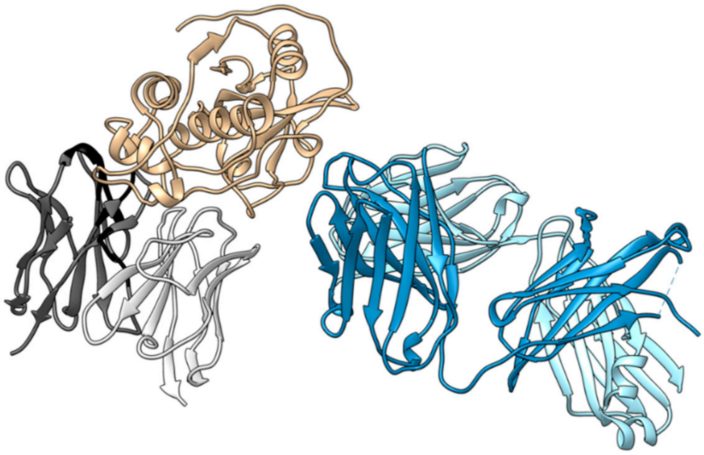

Figure S6. Superimposition of 6A8 scFv – Der f 1 best docking pose and 4C1 Fab – Der f 1 crystal structure. This shows that both antibody fragments recognized different epitope regions of the antigen. 6A8 scFv is colored the same way as Figure 5B. The ribbon of Der f 1 is colored tan and 4C1 heavy chain is in steel blue and light chain is light blue.

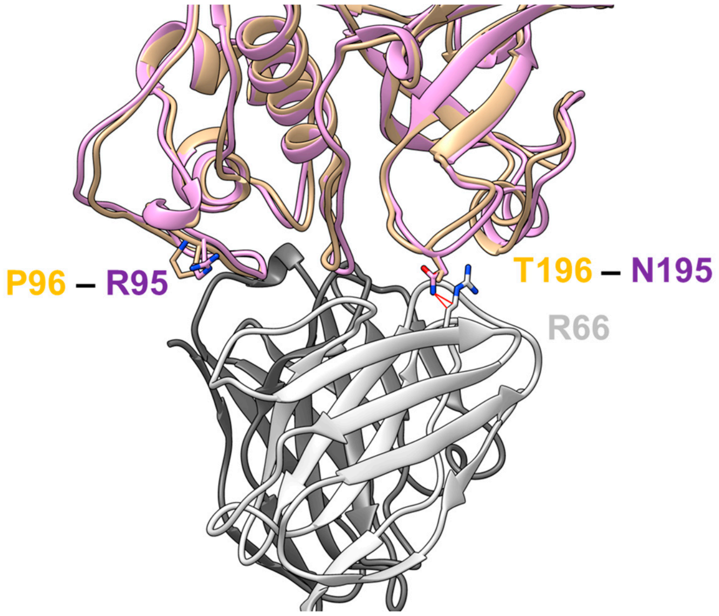

Figure S7. Superimposition of Der p 1 structure (plum color) with the Der f 1 from the top rank docking pose provides insight on the inability of 6A8 to bind to Der p 1. Two main differences are found in the epitope – paratope interface when Der p 1 is present. P^{96} is substituted by R^{95}, which may impact the overall chemical environment of these interactions. N^{195} (instead of T^{196}) causes a direct clash with R^{66} of the light chain (shown in red lines) instead of forming a hydrogen bond like T^{196} does.

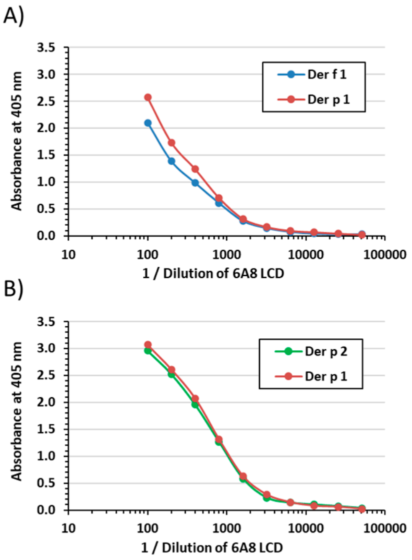

Figure S8. ELISA Binding Assays of 6A8 light chain dimer to Der f 1 and Der p 1 (A) and Der p 2 and Der p 1 (B) allergens. 6A8 light chain displays binding to all three allergens, whereas the complete 6A8 antibody is specific to Der f 1 (Figure S3). Data are duplicates ± SD.

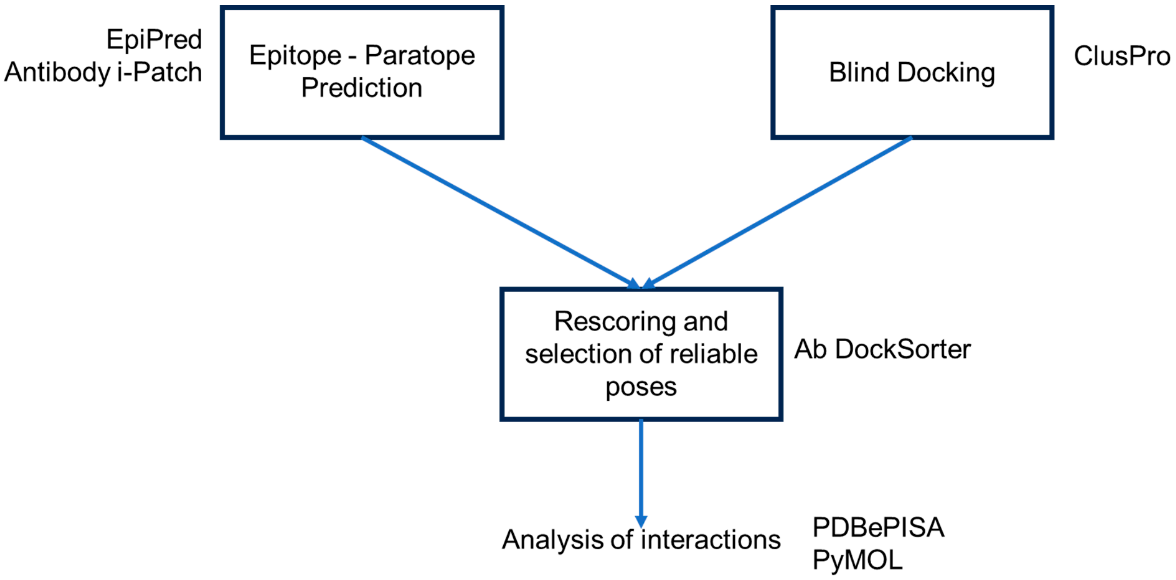

Figure S9. Scheme of the computational approach taken to obtain a robust model of the different protein-protein interactions.
